# Development and validation of a risk prediction model for incident liver cancer

**DOI:** 10.3389/fpubh.2022.955287

**Published:** 2022-09-20

**Authors:** Yingxin Liu, Jingyi Zhang, Weifeng Wang, Guowei Li

**Affiliations:** ^1^Center for Clinical Epidemiology and Methodology, Guangdong Second Provincial General Hospital, Guangzhou, China; ^2^Department of Gastroenterology and Hepatology, Guangdong Second Provincial General Hospital, Guangzhou, China; ^3^Department of Health Research Methods, Evidence, and Impact, McMaster University, Hamilton, ON, Canada

**Keywords:** risk prediction, risk management, screening, nomogram, liver cancer

## Abstract

**Objective:**

We aimed to develop and validate a risk prediction model for liver cancer based on routinely available risk factors using the data from UK Biobank prospective cohort study.

**Methods:**

This analysis included 359,489 participants (2,894,807 person-years) without a previous diagnosis of cancer. We used the Fine-Gray regression model to predict the incident risk of liver cancer, accounting for the competing risk of all-cause death. Model discrimination and calibration were validated internally. Decision curve analysis was conducted to quantify the clinical utility of the model. Nomogram was built based on regression coefficients.

**Results:**

Good discrimination performance of the model was observed in both development and validation datasets, with an area under the curve (95% confidence interval) for 5-year risk of 0.782 (0.748–0.816) and 0.771 (0.702–0.840) respectively. The calibration showed fine agreement between observed and predicted risks. The model yielded higher positive net benefits in the decision curve analysis than considering either all participants as being at high or low risk, which indicated good clinical utility.

**Conclusion:**

A new risk prediction model for liver cancer composed of routinely available risk factors was developed. The model had good discrimination, calibration and clinical utility, which may help with the screening and management of liver cancer for general population in the public health field.

## Introduction

Liver cancer is one of the most common cancers worldwide, ranking the fifth in men and the ninth in women (1). According to World Cancer Research Fund (WCRF), there were over 840,000 new cases in 2018 in 185 countries (1). In the United States, based on the data from Centers for Disease Control and Prevention (CDC) and National Cancer Institute (NCI), 34,638 new cases of liver cancer were reported and 27,685 people died of liver cancer in 2018 (2). Despite the significant advances in therapies, mortality and morbidity of liver cancer remain substantially high, posing a significant public health burden. Preventive strategies including screening, early risk prediction and management are therefore essential for prevention and improved survival of liver cancer (3).

There are several risk prediction models for liver cancer in the literature. Multiple liver cancer risk prediction score systems with emphasis of virological indexes had been proposed previously (3–5). These indexes could markedly increase the specificity of detecting liver cancer; nevertheless, they demanded high economic cost and massive material resources. By contrast, some prediction models were established based on sociodemographic characteristics and clinical factors. However, those models mainly focused on participants with a specific disease or relied heavily on laboratory measures related to liver function (6–10), which compromised their generalizability or applicability. Likewise, prediction model based on genetic risk scores may not be applicable for routine use in the busy practice for the general population (11). There is an urgent need for risk prediction models based on routinely available or easily ascertained factors to help with effective risk prediction and management for liver cancer. Therefore, in this study, we developed, internally validated, and evaluated a risk prediction model for liver cancer using data from the UK Biobank prospective cohort study.

## Materials and methods

### Study population

Details on the UK Biobank study had been described on the website (www.ukbiobank.ac.uk) and in the previous literature (12). In brief, the UK Biobank is a large population-based cohort study with over 0.5 million participants aged 37–73 years enrolled from 2006 to 2010. The study was approved by the North West Multi-Centre Research Ethics Committee. All participants provided written informed consent before enrollment.

Participants with a baseline diagnosis of cancer were excluded from analyses (*n* = 58,741). We included 359,489 participants with complete data on the outcome and potential risk factors. We used the randomized grouping method (seed = 12,345) and made a development/validation split, where the model was fitted to 70% of the participants (*n* = 251,642) and then evaluated on the remaining 30% (*n* = 107,847). The flowchart for our study design is shown in Supplementary Figure 1.

### Variable selection

Baseline data were collected through participants' self-reported questionnaires, interviews with nurses and physical measurements, with hospital in-patient records as supplements. The selection of candidate predictors was based on clinical knowledge and literature reviews. These candidate risk factors included: (1) Sociodemographic factors, including age (years), sex (male or female), residential area (urban or rural) and Townsend deprivation index (TDI); (2) Physical measurement, including body mass index (BMI; kg/m^2^); (3) Lifestyle behaviors, including smoking status (never, previous or current smoker), alcohol drinking status (never, previous or current drinker), consumption of regular vitamin supplements (yes or no) and sleep pattern (healthy, intermediate or poor) (13); (4) Personal medical history (yes or no), including non-viral liver diseases (including cirrhosis, chronic hepatitis and fatty liver), viral hepatitis, diabetes, high cholesterol and cardiovascular disease (CVD); (5) Family medical history (yes or no), including parental history of cancer. Further details of the variables are described in Supplementary Box 1 and Supplementary Table 1.

### Outcome measure

The study outcome was the incidence of liver cancer during follow-up. Liver cancer was referred as malignant neoplasm of liver and intrahepatic bile ducts, and was assessed from cancer registries and hospital in-patient records. The participants were followed up from the date of recruitment (between 2006 and 2010) until the date of diagnosis of liver cancer (ICD-10 code: C22), death or end of follow-up (31 March 2017 for England/Wales, 31 October 2016 for Scotland), whichever occurred first.

### Statistical analysis

For model development, continuous variables were converted into categorical form for easy interpretation and enhanced generalization, which was a general practice in the literature for risk prediction models aiming at prompt use and wide acceptability clinically (14, 15). The cut-off points of those continuous variables were determined according to clinical knowledge and current practice in the literature (8, 14, 16). Each candidate variable was considered for model inclusion if its *P*-value was below 0.2 in the univariate analysis to demonstrate its face validity (Supplementary Table 3). Based on clinical expertise and statistical knowledge, after group discussion we included 12 predictors to construct the prediction model: sex (male or female), age (40–49, 50–59, or ≥60 years) (8, 14), BMI (<25, 25–29.9, or ≥30 kg/m^2^) (16), smoking status (never, previous, or current smoker), drinking status (never, previous, or current drinker), sleep pattern (poor, intermediate, or healthy), family history of cancer (yes or no), diabetes (yes or no), high cholesterol (yes or no), CVD (yes or no), viral hepatitis (yes or no), liver disease (yes or no).

Fine-Gray regression model was performed to estimate the absolute risk of liver cancer, accounting for the competing risk of all-cause death, while data of death were collected from death registry. Five-year risks of liver cancer were computed from the cumulative incidence function (CIF) obtained by the competing risk regression model (17). Subhazard ratio (sHR) and corresponding 95% confidence interval (95% CI) were used to describe the relationship between the predictors and liver cancer risk.

Model discrimination and calibration were validated internally. We used receiver operating characteristic (ROC) curves, area under the curve (AUC) with corresponding 95% CI, and the Somers' D statistics to assess model discrimination (18). Model calibration was measured through plotting the predicted mean risks against the observed risks by a tenth of the predicted risks (19, 20), where the observed risks were calculated by using the Nelson-Aalen method. Nomogram was conducted to generate a user-friendly graphical interface of our model (Supplementary Box 2; Supplementary Figure 3) (21–23).

All analyses were carried out using SAS (SAS/STAT User's Guide, Version 9.4; SAS Institute, Cary, NC) and R (version 4.1.0; The R Foundation, Vienna, Austria). All statistical tests were two-sided, and we considered *P* < 0.05 to be statistically significant. R packages *rms* and *riskRegression* were used for the analysis (24, 25).

### Clinical utility

Decision curve analysis (DCA), a comprehensive method for the assessment of diagnostic tests and prediction models, was conducted to evaluate the clinical utility of our prediction model (21, 22, 26, 27). Net benefit, a key measure in DCA, was computed by weighting the true positive rate minus the false positive rate weighted on the risk threshold. Decision curve plotted the predicted net benefit of the prediction model against assuming all participants at high risk or low risk across all possible risk thresholds. A higher positive net benefit indicated a better clinical utility. R package *dcurves* was employed for the DCA analysis (28). Further details of DCA are described in Supplementary Box 3. The R codes used for the analyses in this study are shown in Supplementary Box 4.

## Results

### Descriptions of participant characteristics

Among the 359,489 included participants (2,894,807 person-years), there were a total of 280 incident liver cancer events (197 in the development and 83 in the validation dataset) and 9,791 deaths (6,947 in the development and 2,844 in the validation dataset) documented. Within the 5-year follow up, there were 159 liver cancer events (113 in the development and 46 in the validation dataset) and 4,494 deaths (3,162 in the development and 1,332 in the validation dataset) found. Supplementary Figure 2 displays the CIF curve of liver cancer during follow-up. Characteristics of the participants are presented in Table 1. There were no statistical differences in baseline characteristics between the development and validation datasets (Supplementary Table 2). When compared with those without liver cancer, participants diagnosed with liver cancer were older, more likely to be males and overweight, had a poorer sleep pattern, and were more likely to have family history of cancer, diabetes, high cholesterol, CVD, viral hepatitis and non-viral liver diseases.

**Table 1:** Description of participant characteristics in the development and validation dataset.

**Characteristic**	**The development dataset**		**The validation dataset**	
	**No liver cancer (*n =* 251,445)**	**Liver cancer** **(*n =* 197)**	**No liver cancer** **(*n =* 107,764)**	**Liver cancer** **(*n =* 83)**
**Age (years)**				
<50	63,069 (25.08)	9 (4.57)	26,953 (25.01)	1 (1.20)
50–59	85,302 (33.92)	55 (27.92)	36,500 (33.87)	28 (33.73)
≥60	103,074 (40.99)	133 (67.51)	44,311 (41.12)	54 (65.06)
**Townsend deprivation index**				
< -2.5	114,933 (45.71)	90 (45.69)	49,147 (45.61)	32 (38.55)
−2.5–0	67,207 (26.73)	56 (28.43)	29,103 (27.01)	24 (28.92)
0–2.4	36,591 (14.55)	27 (13.71)	15,529 (14.41)	14 (16.87)
≥2.5	32,394 (12.88)	24 (12.18)	13,867 (12.87)	13 (15.66)
**Sex**				
Female	134,947 (53.67)	78 (39.59)	57,552 (53.41)	27 (32.53)
Male	116,498 (46.33)	119 (60.41)	50,212 (46.59)	56 (67.47)
**College degree and higher**				
No	166,290 (66.13)	140 (71.07)	71,101 (65.98)	59 (71.08)
Yes	83,138 (33.06)	57 (28.93)	35,796 (33.22)	23 (27.71)
**Ethnicity**				
White	237,650 (94.51)	182 (92.39)	101,833 (94.50)	82 (98.80)
Others	13,133 (5.22)	14 (7.11)	5,611 (5.21)	1 (1.20)
**Residential area**				
Rural	35,741 (14.21)	20 (10.15)	15,250 (14.15)	14 (16.87)
Urban	213,188 (84.79)	176 (89.34)	91,416 (84.83)	67 (80.72)
**BMI (kg/m** ^**2**^ **)**				
<25	83,263 (33.11)	53 (26.90)	35,411 (32.86)	22 (26.51)
25–29.9	107,294 (42.67)	75 (38.07)	46,293 (42.96)	32 (38.55)
≥30	60,888 (24.22)	69 (35.03)	26,060 (24.18)	29 (34.94)
**Physical activity (MET minutes/week)**				
<600	34,392 (13.68)	35 (17.77)	14,655 (13.60)	19 (22.89)
600–3,999	125,246 (49.81)	91 (46.19)	53,978 (50.09)	39 (46.99)
≥4,000	45,256 (18.00)	30 (15.23)	19,267 (17.88)	13 (15.66)
**Smoking status**				
Never	138,327 (55.01)	72 (36.55)	59,513 (55.23)	30 (36.14)
Previous	86,969 (34.59)	102 (51.78)	37,185 (34.51)	42 (50.60)
Current	26,149 (10.40)	23 (11.68)	11,066 (10.27)	11 (13.25)
**Drinking status**				
Never	10,453 (4.16)	8 (4.06)	4,552 (4.22)	5 (6.02)
Previous	8,518 (3.39)	12 (6.09)	3,534 (3.28)	6 (7.23)
Current	232,474 (92.46)	177 (89.85)	99,678 (92.50)	72 (86.75)
**Coffee intake**				
No	55,933 (22.24)	48 (24.37)	23,753 (22.04)	21 (25.30)
Yes	195,512 (77.76)	149 (75.63)	84,011 (77.96)	62 (74.70)
**Vitamin supplement**				
No	171,075 (68.04)	139 (70.56)	73,467 (68.17)	58 (69.88)
Yes	79,551 (31.64)	57 (28.93)	33,937 (31.49)	24 (28.92)
**Mineral supplement**				
No	143,726 (57.16)	125 (63.45)	61,796 (57.34)	48 (57.83)
Yes	107,331 (42.69)	72 (36.55)	45,779 (42.48)	35 (42.17)
**Sleep pattern**				
Poor	5,971 (2.37)	14 (7.11)	2,592 (2.41)	5 (6.02)
Intermediate	98,940 (39.35)	92 (46.70)	42,093 (39.06)	35 (42.17)
Healthy	146,534 (58.28)	91 (46.19)	63,079 (58.53)	43 (51.81)
**Family history of cancer**				
No	174,862 (69.54)	130 (65.99)	75,281 (69.86)	54 (65.06)
Yes	76,583 (30.46)	67 (34.01)	32,483 (30.14)	29 (34.94)
**Diabetes**				
No	234,971 (93.45)	151 (76.65)	100,771 (93.51)	65 (78.31)
Yes	16,474 (6.55)	46 (23.35)	6,993 (6.49)	18 (21.69)
**High cholesterol**				
No	204,958 (81.51)	137 (69.54)	87,736 (81.41)	56 (67.47)
Yes	46,487 (18.49)	60 (30.46)	20,028 (18.59)	27 (32.53)
**CVD**				
No	100,739 (40.06)	45 (22.84)	43,026 (39.93)	16 (19.28)
Yes	150,706 (59.94)	152 (77.16)	64,738 (60.07)	67 (80.72)
**Viral hepatitis**				
No	250,821 (99.75)	184 (93.40)	107,484 (99.74)	81 (97.59)
Yes	624 (0.25)	13 (6.60)	280 (0.26)	2 (2.41)
**Liver disease**				
No	250,924 (99.79)	181 (91.88)	107,552 (99.80)	75 (90.36)
Yes	521 (0.21)	16 (8.12)	212 (0.20)	8 (9.64)

BMI, Body mass index; MET, Metabolic Equivalent of Task; CVD, Cardiovascular disease.

### Development of the risk prediction model

The sHR (95% CI) from the multivariable Fine-Gray competing risk prediction model in the development dataset are listed in Table 2. It was found that males, older age (50–59, and ≥60), previous smoking status, diabetes, viral hepatitis and non-viral liver diseases were significantly associated with increased risk of liver cancer, with sHR ranging from 1.54 to 16.83 (all *P* < 0.05). A better sleep pattern (intermediate, and healthy) was significantly associated with decreased risk of liver cancer (*P* < 0.05). High cholesterol was non-significantly associated with a decreased risk of liver cancer (sHR = 0.76, 95% CI: 0.51–1.13), while it was significantly related with increased risk of liver cancer from the univariate analysis (sHR = 1.99, 95% CI: 1.55–2.56; Supplementary Table 3).

**Table 2:** Subhazard ratios and 95% confidence intervals for the predictors included in the multivariable Fine-Gray regression model.

**Predictors**	**sHR**	**95% CI**	***P*-value**
**Male sex**	1.54	1.15, 2.06	0.004
**Age**
<50	Ref	-	-
50–59	3.95	1.93, 8.07	<0.001
≥60	7.52	3.73, 15.20	<0.001
**BMI**
<25	Ref	-	-
25–29.9	0.80	0.56, 1.15	0.232
≥30	1.04	0.70, 1.56	0.844
**Smoking status**
Never	Ref	-	-
Previous	1.68	1.23, 2.31	0.001
Current	1.51	0.94, 2.41	0.085
**Drinking status**
Never	Ref	-	-
Previous	1.04	0.42, 2.60	0.933
Current	1.02	0.48, 2.15	0.963
**Sleep pattern**
Poor	Ref	-	-
Intermediate	0.53	0.29, 0.95	0.033
Healthy	0.41	0.22, 0.75	0.004
**Family history of cancer**	1.15	0.86, 1.54	0.355
**Diabetes**	3.10	2.05, 4.69	<0.001
**High cholesterol**	0.76	0.51, 1.13	0.170
**CVD**	1.31	0.91, 1.87	0.145
**Viral hepatitis**	16.83	8.92, 31.76	<0.001
**Liver disease**	15.46	8.29, 28.86	<0.001

sHR, Subhazard Ratio; CI, Confidence Interval; BMI, Body Mass Index; CVD, Cardiovascular disease.

### Performance of the model

**Figure 1:**
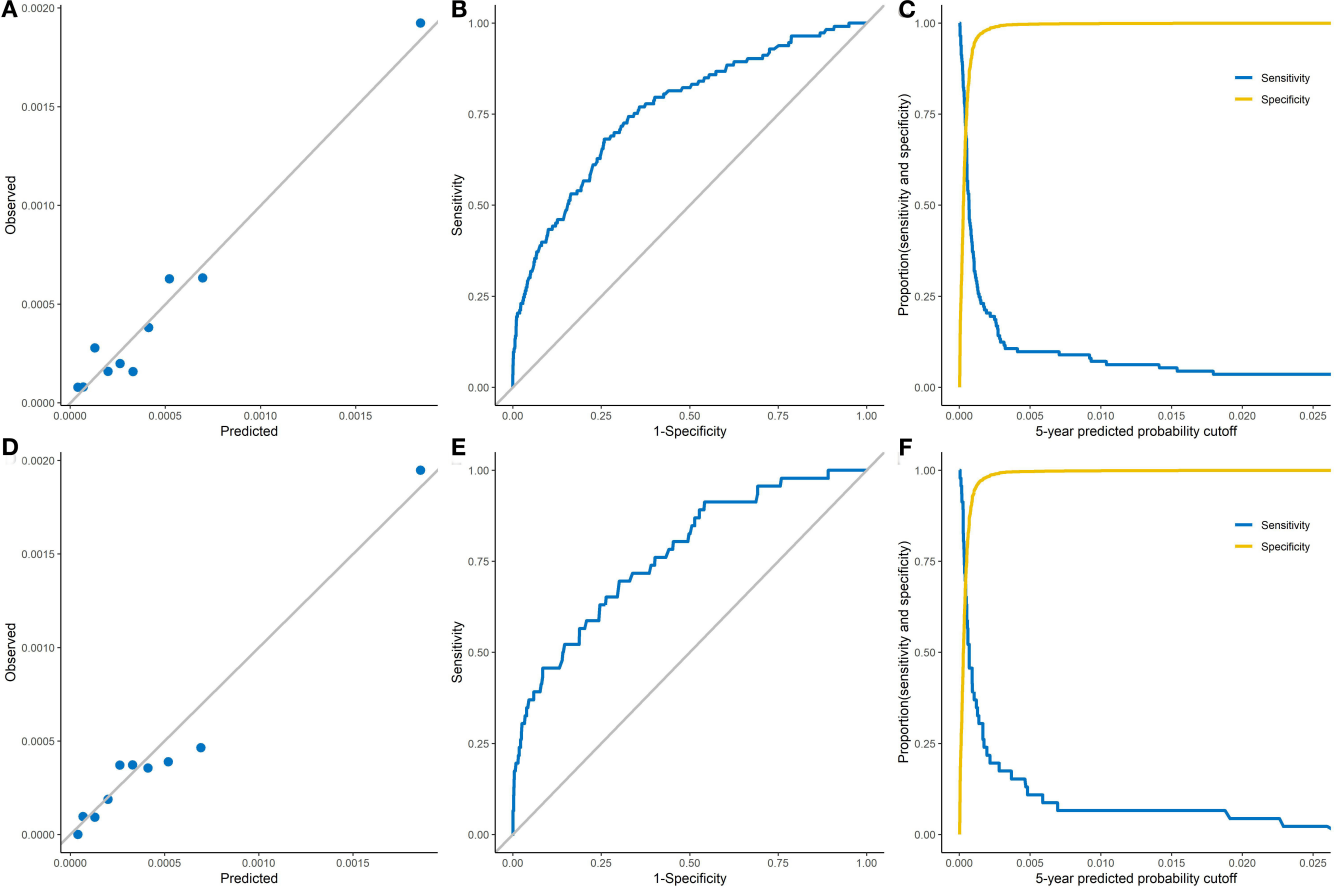
The calibration and discrimination of the Fine-Gray model in both development and validation datasets. **(A)**, The observed 5-year probability of liver cancer of the development dataset. **(B)**, Receiver operating characteristic curve of the development dataset (AUC: 0.782, 95% CI: 0.748–0.816). **(C)**, Sensitivity and specificity on the basis of the predicted probability cutoff of the development dataset. **(D)**, The observed 5-year probability of liver cancer of the validation dataset. **(E)**, Receiver operating characteristic curve of the validation dataset (AUC: 0.771, 95% CI: 0.702–0.840). **(F)**, Sensitivity and specificity on the basis of the predicted probability cutoff of the validation dataset.

The calibration and discrimination performances are displayed in Figure 1. According to the calibration plots in Figure 1A (the development dataset) and Figure 1D (the validation dataset), the observed 5-year probabilities agreed well with the predicted 5-year risks, which indicated that the risk prediction model was well calibrated.

The ROC curves for the prediction model are shown in Figure 1B (the development dataset) and Figure 1E (the validation dataset). The sensitivity and specificity of the model are plotted by the 5-year predicted probability in Figure 1C (the development dataset) and Figure 1F (the validation dataset). Within 5-years of follow-up, the prediction model had an AUC of 0.782 (95% CI: 0.748–0.816) and Somers' D statistics of 0.563 in the development dataset (Table 3). Results from internal validation showed an AUC of 0.771 (95% CI: 0.702 - 0.840) and Somers' D statistics of 0.541 in the validation dataset. In the development dataset, the maximum Youden index of 0.42 was identified to reach the best discrimination performance, which corresponded to a risk threshold of 51.3 per 100,000, a specificity of 74.1% and a sensitivity of 68.1% (Table 3). A maximum Youden index of 0.40 in the validation dataset would yield a risk threshold of 46.0 per 100,000, a specificity of 70.0% and a sensitivity of 69.6%.

**Table 3:** Performance of the prediction model for liver cancer within 5 years of follow-up in both development and validation datasets.

**Dataset**	**AUC (95% CI)**	**Youden index**	**Risk threshold per 100,000**	**Sensitivity (%)**	**Specificity (%)**	**Somers' D**
Development	0.782 (0.748–0.816)	0.42	51.3	68.1	74.1	0.563
Validation	0.771 (0.702–0.840)	0.40	46.0	69.6	70.0	0.541

AUC, area under the curve; CI, confidence interval.

The nomogram of our prediction model is shown in Supplementary Figure 3. The score points of the nomogram are displayed in Supplementary Table 4, while Supplementary Table 5 demonstrates the 5-year risks of liver cancer corresponding to the total score points.

### DCA for clinical utility

**Figure 2:**
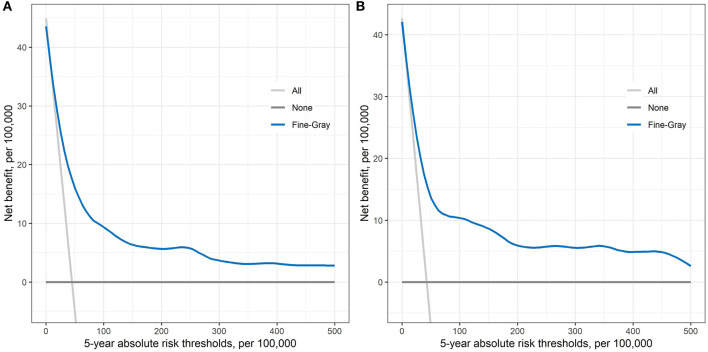
Decision curve obtained from plotting the net benefit of detecting liver cancer at different 5-year absolute risk thresholds in both development dataset **(A)** and validation dataset **(B)**.

Figure 2 shows the net benefit curves for the prediction model within 5-years of follow-up in both development and validation datasets. The horizontal axis is the 5-year risk threshold used to define high risk, while the vertical axis is the net benefit at the current risk threshold. We compared the prediction model with the extreme strategies of assuming all or none participants at high risk. The analysis showed that the prediction model yielded a higher positive net benefit than all the other alternatives across 5-year absolute risk thresholds ranging from 0 to 500 per 100,000.

## Discussion

Based on data from the large-scale prospective cohort study, we developed and internally validated a risk prediction model for the absolute 5-year cumulative risk of liver cancer. The prediction model showed good calibration and discrimination, and had a good clinical utility.

The risk prediction model was composed of predictors that were routinely available in clinical practice. As expected, male sex, old age, previous smoking status, diabetes, viral hepatitis and non-viral liver diseases were significantly associated with increased risk of liver cancer. Viral hepatitis and non-viral liver diseases that were typical disorders related to liver function, could substantially rise the risk of liver cancer. Data on them could be easily collected from patients' health records or self-report in the general population. Given the large HRs for the relationship between viral hepatitis and non-viral liver diseases and liver cancer risk, we conducted a *post-hoc* multivariable Fine-Gray model without these two factors to assess the prediction ability of other variables. The new prediction model had an AUC of 0.746 (95% CI: 0.712–0.780) in the development dataset, which did not significantly differ from the original model with an AUC of 0.782 (95% CI: 0.748–0.816). Therefore, our model was robust even with the presence of viral hepatitis and liver diseases that were highly related with liver cancer risk.

Our study showed that a healthy sleep pattern was significantly related to decreased risk of liver cancer, which was consistent with previous observational studies (29). For example, one study showed that sleep quality predicted up to 20.4% of the variability of liver stiffness after adjusting for potential confounders (30). Compelling evidence reported that sleep insufficiency had significant effects including reduction of leptin and elevation of ghrelin, which might predispose to liver diseases by means of proinflammatory markers and stress response (30). Baseline high cholesterol was non-significantly associated with decreased risk of liver cancer in our model, which may be due to at least in part, the cholesterol-lowering medications used in those with high cholesterol. Being effective in preventing CVD morbidity and mortality, the use of statins had been shown to inversely relate to risk of various cancers (31). For instance, a meta-analysis reported that the use of statins was significantly associated with reduced risk of liver cancer in those taking statins for CVD prevention (RR = 0.58, 95% CI: 0.51 - 0.67) (32). Moreover, previous clinical research indicated that statins possessed synergism with other therapeutic agents *in vitro* and *in vivo* for liver cancer (32). Another study indicated that statins might involve the ubiquinone inhibition, which might subsequently lead to the apoptosis of preneoplastic liver cells (31). Nevertheless, the relationship between high cholesterol and decreased risk of liver cancer required more high-quality research for further exploration and validation.

### Comparison with previous studies

There had been multiple liver cancer risk prediction models in the literature (3–8). Previous risk score systems mainly focused on virological indexes to increase accuracy of detecting liver cancer; however this would cost healthcare resources and impose laboratory burden especially in under-resourced areas (3–5). Furthermore, these prediction systems were exclusively limited to HBV or HCV carriers, which would significantly limit their application to the general population. By contrast, several risk prediction models had been established based on sociodemographic characteristics and metabolic indexes for the general population (6, 7). Nevertheless, the previous study targeted on participants with diabetes and incorporated complex algorithms of machine learning, which would therefore compromise its intake and application in real-world clinical practice (6). Although the Taiwan study aimed for the general average-risk population, the corresponding cohort was constructed from participants engaged in a medical screening program who had an above-average socioeconomic status (7). Moreover, it unduly emphasized laboratory measures for assessment of the current liver function, generating difficulty in application in general population and busy clinical practice.

One recent observational research based on data from the China Kadoorie Biobank (CKB) Study with 0.5 million participants was published to build a personalized risk prediction model for 10-year liver cancer risk (CKB-PLR) (8). The CKB-PLR model was composed of sociodemographic data, lifestyle characteristics and blood biochemistry measures. However, complicated mathematical incorporation in the CKB-PLR model would jeopardize its applicability for physicians and patients. Quantitative values of predictors including physical activity and random glucose were required in the model, which was challenging for measurement in busy clinical practice. Moreover, the use of random glucose in the CKB-PLR model was inappropriate given its substantial variability affected by multiple conditions. Inclusion of random glucose may provide a snapshot of the instant glucose level at best, and may be misleading or even incur biased results when building the prediction model.

In our study, we focused on established factors of liver cancer that were routinely available or easily captured. Furthermore, each variable was grouped into categorical form in the model to maximize its prompt use and easy intake. We also generated the nomogram and performed DCA to improve the straightforward visualization and clinical applicability for our model. Thus, if externally validated, our model had the potential to be implemented in clinical practice for quick risk prediction and decision-making.

## Strength and limitations

This study has several strengths. First, we used high-quality data from the large-scale UK Biobank cohort for model building and evaluation, while the UK Biobank had already been used for risk prediction models for cancers (8, 11, 14, 33). Second, competing risk bias was adequately address by using the competing risk model given the long follow-up in the cohort. Third, the inclusion of sleep pattern in the model was the first for liver cancer risk prediction, to the best of our knowledge. Forth, our endeavors for simplicity and straightforwardness would enhance the model applicability in real-world settings.

There are several limitations to this study. Initially, we were unable to externally validate the proposed risk prediction model due to lack of external data, although internal validation was conducted with robust findings reported. Furthermore, direct comparison with other models is difficult due to differences in study design, predictor definitions, and risk factor patterns in the study populations. External validation of our model, and comparisons with other clinical features or existed prediction tools would be worthwhile endeavors to further justify the validity and applicability of our model, especially for high-risk population (3–7). Second, the study participants were dominantly from European descent, which might affect the generalizability of the model to other populations with different average risks (1). Our study reported a relatively low incidence of liver cancer of 0.078% (280/359,489), which was in line with some epidemiological studies based on large-scale datasets that reported an incidence of liver cancer ranging from 0.041 to 0.5% (8, 34, 35). Furthermore, even though recognizing the importance of other potential predictors including physical activity and medication intake, they were not selected for model development to maximize its applicability in practice, which may impair the predictive validity of our prediction model. Besides, we only used baseline data for model building and did not consider data on temporal changes, which might also affect the model performance. Moreover, subtypes of liver cancer were not considered in our study due to insufficiency of incident events.

## Conclusion

In conclusion, we developed and internally validated a liver cancer risk prediction model based on routinely available data, using data of the UK Biobank study. The prediction model had acceptable calibration and discrimination, and a good clinical utility. If externally validated, the model had the potential to be used in clinical practice for liver cancer screening, risk prediction and management in the public health field.

## Data availability statement

Publicly available datasets were analyzed in this study. This data can be found here: This research has been conducted using the UK Biobank Resource under Application Number 63844. Data are available from the UK Biobank (https://www.ukbiobank.ac.uk/) for researchers who meet the criteria and gain approvals to access the research database from the UK Biobank access management committee at the University of Oxford.

## Ethics statement

The studies involving human participants were reviewed and approved by the North West Multi-Centre Research Ethics Committee (Ref No. 11/NW/0382). The patients/participants provided their written informed consent to participate in this study.

## Author contributions

YL, JZ, WW, and GL: conceived and designed the study, acquired data, and conducted statistical analyses and interpretation. YL and GL: drafted the manuscript, and provided statistical support. JZ and WW: provided professional support, and made several critical revisions to the manuscript. All authors read and approved the final manuscript.

## Funding

This study was funded by the National Natural Science Foundation of China (No. 82103906), and the Science Foundation of Guangdong Second Provincial General Hospital (No. YY2018-002).

## Conflict of interest

The authors declare that the research was conducted in the absence of any commercial or financial relationships that could be construed as a potential conflict of interest.

## Publisher's note

All claims expressed in this article are solely those of the authors and do not necessarily represent those of their affiliated organizations, or those of the publisher, the editors and the reviewers. Any product that may be evaluated in this article, or claim that may be made by its manufacturer, is not guaranteed or endorsed by the publisher.
