# Supplementary material for: Development and validation of a risk prediction model for incident liver cancer
Source: Front Public Health. 2022 Sep 20;10:955287. doi: 10.3389/fpubh.2022.955287 (PMC9768800; doi:10.3389/fpubh.2022.955287)
Supplement: Supplementary file 1 [file Data_Sheet_1.docx]

**Supplemental Box 1.** Definition of sleep pattern

We adopted the algorithm to build the sleep pattern based on five sleep factors from a previous study using the UK Biobank study ^1^. The score of each factor was 0, or 1 when low-risk responses were got as follows: no insomnia (responses as “never/rarely”), no excessive daytime sleepiness (“never/rarely” or “sometimes”), no habitual snoring (“no”), early chronotype (a “morning” person, or more a “morning” than “evening” person), and adequate sleep duration (7-8 hours per day). Based on the sum of five scores, the sleep pattern was then defined as three levels, including “healthy sleep pattern” (summed score $\geq$ 4), “intermediate sleep pattern” (2 $\leq$ summed score $\leq$ 3), and “poor sleep pattern” (summed score $\leq$ 1).

**Reference:**

1. Fan M, Sun D, Zhou T, et al. Sleep patterns, genetic susceptibility, and incident cardiovascular disease: a prospective study of 385,292 UK biobank participants. Eur Heart J 2020;41(11):1182-89.

**Supplemental Box 2.** Description of Nomogram

Nomogram was conducted as a user-friendly interface of our model, allowing for visual and easy intake for clinical decision-making ^1^.

Each estimated coefficient parameter (nature logarithm of sHR) was reweighted by dividing the total coefficient estimates, multiplying by 100, and then rounding to the nearest integer. The consequent point of each variable had a minimum value of 0 (the lowest risk) and a maximum value of 100 (the highest liver cancer risk). The 5-year risks of liver cancer corresponding to the total score points were also estimated from the Fine-Gray regression model.

**Reference:**

1. Iasonos A, Schrag D, Raj GV, Panageas KS. How to build and interpret a nomogram for cancer prognosis. J Clin Oncol. 2008;26(8):1364-70.

**Supplemental Box 3.** Decision curve analysis to estimate the clinical utility of the multivariable Fine-Gray regression model

Decision curve analysis (DCA) was conducted to assess the clinical utility of the prediction model^1,2^. Net benefit, acting as a key measure in DCA, was computed by weighting the true positive rate minus the false positive rate weighted on the risk threshold, which puts benefits and harms on the same scale.

$$Net benefit=\frac{TP-FP*\left( \frac{p}{1-p} \right)}{N}$$

Where *N* is the total sample size of participants, *TP* indicates true positive counts, *FP* indicates false positive counts, *p* indicates the risk threshold. In our case, $\frac{p}{1-p}$ indicates the ratio of the harm of unnecessary liver cancer examination versus the benefit of necessary examination for participants with liver cancer.

Decision curve plots the predicted net benefit of the prediction model against assuming all participants at high risk or low risk across all possible risk thresholds. Higher positive net benefit indicates better clinical utility.

**References:**

1. Vickers AJ, Elkin EB. Decision curve analysis: a novel method for evaluating prediction models. Med Decis Making 2006;26(6):565-74.
2. Vickers AJ, Cronin AM, Elkin EB, Gonen M. Extensions to decision curve analysis, a novel method for evaluating diagnostic tests, prediction models and molecular markers. BMC Med Inform Decis Mak 2008;8:53

**Supplemental Box 4.** R codes used in the analyses for this study

##formula

fmla.variable<-

"Sex+Age_level+BMI_level+Smoking_status+Drinking_status+Sleep_pattern+

Family_cancer+Diabetes+Cholesterol+CVD+Viral_hepatitis+Liver_disease"

fmla <- as.formula(

paste("Hist(diagnosis_liver_cancer_time, status) ~",

fmla.variable))

surv.start.stop.fmla <- as.formula(

paste("Surv(fgstart,fgstop,fgstatus) ~",

fmla.variable))

##converted data to Fine-Gray form

pdata <- finegray(Surv(diagnosis_liver_cancer_time, factor(status)) ~ ., data=dftrain)

pdata$Time<-pdata$fgstop-pdata$fgstart

dd <- datadist(pdata) #

options(datadist='dd') #

##Fine-Gray1

fit<- cph(surv.start.stop.fmla,data=pdata,weight=fgwt,x=T,y=T,surv=T)

#nomogram

surv<-Survival(fit)

nom <- nomogram(fit,

fun=list(function(x) 1-surv(1825, x)),

fun.at =c(0.00002, 0.00005, 0.0001, 0.0002, 0.0005, 0.001, 0.002, 0.005, 0.01, 0.02, 0.05, 0.1, 0.2, 0.5),

funlabel=c("5-Year Event Probability"),

abbrev = F

)

##Fine-Gray2

fgr <- FGR(fmla,data=dftrain,cause=1)

pred.fgr.train<-predictRisk(fgr,newdata=dftrain, times = c(365*5), cause = 1)

pred.fgr.test<-predictRisk(fgr,newdata=dftest, times = c(365*5), cause = 1)

##DCA-train

dftrain$pred.fgr<-pred.fgr.train

gg_dca <-

dca(Surv(diagnosis_liver_cancer_time,factor(status))~pred.fgr,

data = dftrain,

thresholds = seq(0, 0.005, by = 0.00005),

time=1825,

label = list(pred.fgr='Fine-Gray',

all='All',

none='None')) %>%

plot(smooth = TRUE, show_ggplot_code = TRUE)

#ROC-train

score<-Score(list("Fine-Gray"=fgr),

formula = Hist(diagnosis_liver_cancer_time,status)~1,

data=dftrain,

times =365*5,

cause=1,

se.fit=F,

plots = c("calibration","roc"),

metrics=c("auc","brier"),

summary = "risks")

##########ggplot####################################################

##calibration

gg.cali1<-ggplot(data=p.cali$plotFrames$'Fine-Gray',

mapping = aes(x =Pred, y = Obs,color=factor(1))) +

geom_point(size=3)+

theme_classic()+

theme(legend.position="None")+

labs(x="Predicted", y="Observed")+

geom_abline(intercept = 0, slope = 1, col="grey",size=1)

##ROC

gg.roc1<-ggplot(data=score$ROC$plotframe,mapping = aes(x = FPR, y = TPR,color=factor(1))) +

geom_line(size=1.2,linetype=1)+

theme_classic()+

theme(legend.position="None")+

coord_cartesian(xlim=c(0,1),ylim = c(0,1))+

labs(x="1-Specificity", y="Sensitivity")+

geom_abline(intercept = 0, slope = 1, col="grey",size=1)

**Supplemental Figure 1.** Flowchart for the study design


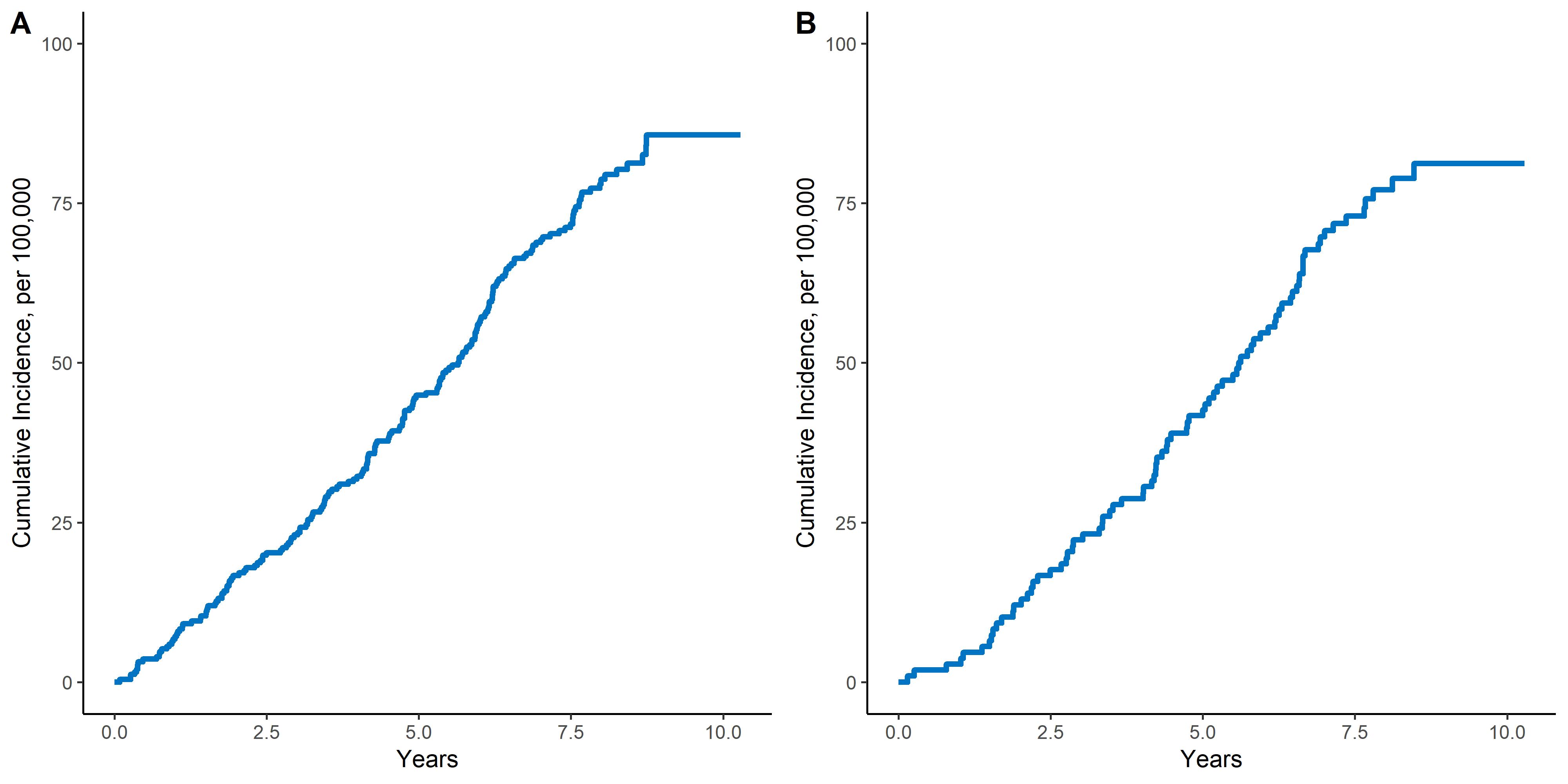


**Supplemental Figure 2.** Cumulative Incidence curves of liver cancer in both development dataset (A) and validation dataset (B).


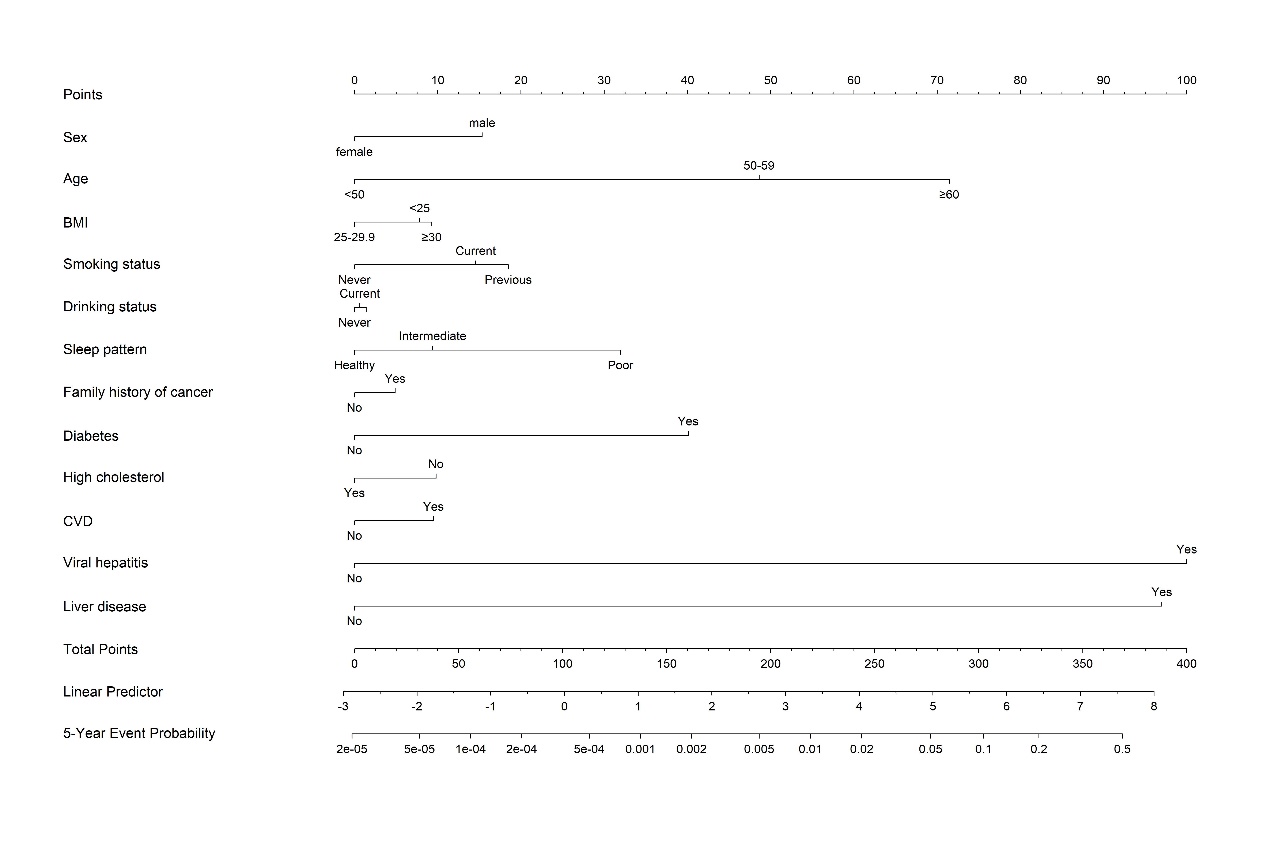


**Supplemental Figure 3**. Nomogram for the multivariable Fine-Gray regression model

**Supplemental Table 1.** Ascertainment of Baseline Characteristics

| **Characteristics** | **Source** | **Code** |
| --- | --- | --- |
| **Liver disease** | HES: ICD10 | K73-K74,K760 |
|  | HES: ICD9 | 5710,5714-5716 |
|  | Baseline: non-cancer illness code | 1158,1506,1604 |
| **Viral hepatitis** | HES: ICD10 | B18 |
|  | HES: ICD9 | 070 |
|  | Baseline: non-cancer illness code | 1156,1578-1582 |
| **Diabetes** | HES: ICD10 | E10-E14 |
|  | HES: ICD9 | 250 |
|  | Baseline: diabetes (2443) | 1 |
|  | Baseline: Medications for diseases (6153) | 3 |
|  | Baseline: Medications for diseases (6177) | 3 |
|  | Baseline: non-cancer illness code | 1220-1223,1521 |
| **CVD** | HES: ICD10 | I10-I15,I20-I25,I26-I28,I30-I52, I60-I69,I70-I79,I80-I89,I95-I99 |
|  | HES: ICD9 | 401-459 |
|  | Baseline: Vascular/heart problems (6150) | 1-4 |
|  | Baseline: non-cancer illness code | 1065-1068,1072-1083,1086-1088,1093-1094,1425-1426,1471,1473,1479,1483-1495,1583-1593 |
| **High cholesterol** | HES: ICD10 | E780 |
|  | HES: ICD9 | 250 |
|  | Baseline: Medications for diseases (6153) | 1 |
|  | Baseline: Medications for diseases (6177) | 1 |
|  | Baseline: non-cancer illness code | 1473 |
| **Family history of cancer** | Baseline: illnesses of parents (20107,20110) | 3,4,5,7 |
|  | HES: ICD10 | Z80 |
|  | HES: ICD9 | V16 |
| **Sleep pattern** | Baseline: Sleep duration (1160) | (7~8)=1 else=0 |
|  | Baseline: Chronotype (1180) | (1,2)=1 else=0 |
|  | Baseline: Sleeplessness (1200) | (1,2)=1 else=0 |
|  | Baseline: Snoring (1210) | (2)=1 else=0 |
|  | Baseline: Daytime dozing (1220) | (0,1)=1 else=0 |

Abbreviations: CVD = Cardiovascular disease; HES = Hospital Episodes Statistics; ICD = International Classification of Disease.

**Supplemental Table 2.** Differences of participant characteristics between the development and validation datasets

| **Characteristic** | **Overall (n=359489)** | **Development Dataset (n=251642)** | **Validation Dataset (n=107847)** | ***P*-value** |
| --- | --- | --- | --- | --- |
| **Age (years)** |  |  |  |  |
| <50 | 90032 (25.04) | 63078 (25.07) | 26954 (24.99) | 0.779 |
| 50-59 | 121885 (33.91) | 85357 (33.92) | 36528 (33.87) |  |
| ≥60 | 147572 (41.05) | 103207 (41.01) | 44365 (41.14) |  |
| **BMI (kg/m2)** |  |  |  |  |
| <25 | 118749 (33.03) | 83316 (33.11) | 35433 (32.85) | 0.227 |
| 25-29.9 | 153694 (42.75) | 107369 (42.67) | 46325 (42.95) |  |
| ≥30 | 87046 (24.21) | 60957 (24.22) | 26089 (24.19) |  |
| **TDI** |  |  |  |  |
| <-2.5 | 164202 (45.68) | 115023 (45.71) | 49179 (45.60) | 0.335 |
| -2.5-0 | 96390 (26.81) | 67263 (26.73) | 29127 (27.01) |  |
| 0-2.4 | 52161 (14.51) | 36618 (14.55) | 15543 (14.41) |  |
| ≥2.5 | 46298 (12.88) | 32418 (12.88) | 13880 (12.87) |  |
| **Sex** |  |  |  |  |
| Female | 192604 (53.58) | 135025 (53.66) | 57579 (53.39) | 0.141 |
| Male | 166885 (46.42) | 116617 (46.34) | 50268 (46.61) |  |
| **College degree or higher** |  |  |  |  |
| No | 237590 (66.09) | 166430 (66.14) | 71160 (65.98) | 0.373 |
| Yes | 119014 (33.11) | 83195 (33.06) | 35819 (33.21) |  |
| **Ethnicity** |  |  |  |  |
| White | 339747 (94.51) | 237832 (94.51) | 101915 (94.50) | 0.820 |
| Others | 18759 (5.22) | 13147 (5.22) | 5612 (5.20) |  |
| **Area** |  |  |  |  |
| Rural | 51025 (14.19) | 35761 (14.21) | 15264 (14.15) | 0.669 |
| Urban | 304847 (84.80) | 213364 (84.79) | 91483 (84.83) |  |
| **Physical activity (MET minutes/week)** |  |  |  |  |
| <600 | 49101 (13.66) | 34427 (13.68) | 14674 (13.61) | 0.365 |
| 600-3999 | 179354 (49.89) | 125337 (49.81) | 54017 (50.09) |  |
| ≥4000 | 64566 (17.96) | 45286 (18.00) | 19280 (17.88) |  |
| **Smoking status** |  |  |  |  |
| Never | 197942 (55.06) | 138399 (55.00) | 59543 (55.21) | 0.370 |
| Previous | 124298 (34.58) | 87071 (34.60) | 37227 (34.52) |  |
| Current | 37249 (10.36) | 26172 (10.40) | 11077 (10.27) |  |
| **Drinking status** |  |  |  |  |
| Never | 15018 (4.18) | 10461 (4.16) | 4557 (4.23) | 0.179 |
| Previous | 12070 (3.36) | 8530 (3.39) | 3540 (3.28) |  |
| Current | 332401 (92.46) | 232651 (92.45) | 99750 (92.49) |  |
| **Coffee intake** |  |  |  |  |
| No | 79755 (22.19) | 55981 (22.25) | 23774 (22.04) | 0.183 |
| Yes | 279734 (77.81) | 195661 (77.75) | 84073 (77.96) |  |
| **Vitamin supplement** |  |  |  |  |
| No | 244739 (68.08) | 171214 (68.04) | 73525 (68.18) | 0.401 |
| Yes | 113569 (31.59) | 79608 (31.64) | 33961 (31.49) |  |
| **Mineral supplement** |  |  |  |  |
| No | 205695 (57.22) | 143851 (57.16) | 61844 (57.34) | 0.289 |
| Yes | 153217 (42.62) | 107403 (42.68) | 45814 (42.48) |  |
| **Sleep pattern** |  |  |  |  |
| Poor | 8582 (2.39) | 5985 (2.38) | 2597 (2.41) | 0.247 |
| Intermediate | 141160 (39.27) | 99032 (39.35) | 42128 (39.06) |  |
| Healthy | 209747 (58.35) | 146625 (58.27) | 63122 (58.53) |  |
| **Family history of cancer** |  |  |  |  |
| No | 250327 (69.63) | 174992 (69.54) | 75335 (69.85) | 0.062 |
| Yes | 109162 (30.37) | 76650 (30.46) | 32512 (30.15) |  |
| **Depression** |  |  |  |  |
| No | 304461 (84.69) | 213168 (84.71) | 91293 (84.65) | 0.649 |
| Yes | 55028 (15.31) | 38474 (15.29) | 16554 (15.35) |  |
| **Diabetes** |  |  |  |  |
| No | 335958 (93.45) | 235122 (93.44) | 100836 (93.50) | 0.482 |
| Yes | 23531 (6.55) | 16520 (6.56) | 7011 (6.50) |  |
| **High cholesterol** |  |  |  |  |
| No | 292887 (81.47) | 205095 (81.50) | 87792 (81.40) | 0.489 |
| Yes | 66602 (18.53) | 46547 (18.50) | 20055 (18.60) |  |
| **CVD** |  |  |  |  |
| No | 143826 (40.01) | 100784 (40.05) | 43042 (39.91) | 0.434 |
| Yes | 215663 (59.99) | 150858 (59.95) | 64805 (60.09) |  |
| **Viral hepatitis** |  |  |  |  |
| No | 358570 (99.74) | 251005 (99.75) | 107565 (99.74) | 0.676 |
| Yes | 919 (0.26) | 637 (0.25) | 282 (0.26) |  |
| **Liver disease** |  |  |  |  |
| No | 358732 (99.79) | 251105 (99.79) | 107627 (99.80) | 0.600 |
| Yes | 757 (0.21) | 537 (0.21) | 220 (0.20) |  |

Abbreviations: BMI = Body Mass Index; TDI = Townsend Deprivation Index; MET= Metabolic Equivalent of Task; CVD = Cardiovascular disease

**Supplemental Table 3.** Subhazard ratios and 95% confidence intervals for the characteristics from the univariate Fine-Gray regression model

| **Characteristic** | **sHR** | **95% CI** | ***P*-value** |
| --- | --- | --- | --- |
| **Male sex** | 1.92 | 1.51,2.45 | <0.001 |
| **Age** | 1.10 | 1.08,1.12 | <0.001 |
| **TDI** | 1.02 | 0.98,1.05 | 0.440 |
| **Non-white ethnicity** | 1.07 | 0.63,1.79 | 0.810 |
| **Urban area** | 1.20 | 0.84,1.72 | 0.320 |
| **BMI** | 1.05 | 1.03,1.07 | <0.001 |
| **Physical activity** |  |  |  |
| <600 | Ref | - | - |
| 600-3999 | 0.661 | 0.48,0.91 | 0.011 |
| ≥4000 | 0.608 | 0.41,0.91 | 0.015 |
| **Smoking status** |  |  |  |
| Never | Ref | - | - |
| Previous | 2.25 | 1.75,2.90 | <0.001 |
| Current | 1.77 | 1.20,2.60 | 0.004 |
| **Drinking status** |  |  |  |
| Never | Ref | - | - |
| Previous | 1.72 | 0.84,3.50 | 0.140 |
| Current | 0.86 | 0.49,1.50 | 0.600 |
| **Sleep pattern** |  |  |  |
| Poor | Ref | - | - |
| Intermediate | 0.41 | 0.25,0.66 | <0.001 |
| Healthy | 0.29 | 0.18,0.47 | <0.001 |
| **Coffee intake** | 0.87 | 0.67,1.15 | 0.330 |
| **Vitamin supplement** | 0.89 | 0.69,1.15 | 0.360 |
| **Family history of cancer** | 1.20 | 0.94,1.53 | 0.150 |
| **Diabetes** | 4.26 | 3.22,5.63 | <0.001 |
| **High cholesterol** | 1.99 | 1.55,2.56 | <0.001 |
| **CVD** | 2.41 | 1.81,3.20 | <0.001 |
| **Viral hepatitis** | 21.73 | 12.90,36.59 | <0.001 |
| **Liver disease** | 44.88 | 29.53,68.23 | <0.001 |

Abbreviations: sHR = Subhazard Ratio; CI = Confidence Interval; TDI = Townsend Deprivation Index; BMI = Body Mass Index; CVD = Cardiovascular disease

**Supplemental Table 4.** The score points of the nomogram estimated from the multivariable Fine-Gray regression model

| **Characteristic** | **Coef** | **sHR** | **95% CI** | ***P*-value** | **Score point** |
| --- | --- | --- | --- | --- | --- |
| **Sex** |  |  |  |  |  |
| Female | Ref | - | - | - | 0 |
| Male | 0.43 | 1.54 | 1.15, 2.06 | 0.004 | 15 |
| **Age** |  |  |  |  |  |
| <50 | Ref | - | - | - | 0 |
| 50-59 | 1.37 | 3.95 | 1.93, 8.07 | <0.001 | 49 |
| ≥60 | 2.01 | 7.52 | 3.73, 15.20 | <0.001 | 71 |
| **BMI** |  |  |  |  |  |
| <25 | Ref | - | - | - | 8 |
| 25-29.9 | -0.22 | 0.80 | 0.56, 1.15 | 0.232 | 0 |
| ≥30 | 0.04 | 1.04 | 0.70, 1.56 | 0.844 | 9 |
| **Smoking status** |  |  |  |  |  |
| Never | Ref | - | - | - | 0 |
| Previous | 0.52 | 1.68 | 1.23, 2.31 | 0.001 | 18 |
| Current | 0.41 | 1.51 | 0.94, 2.41 | 0.085 | 15 |
| **Drinking status** |  |  |  |  |  |
| Never | Ref | - | - | - | 0 |
| Previous | 0.04 | 1.04 | 0.42, 2.60 | 0.933 | 1 |
| Current | 0.02 | 1.02 | 0.48, 2.15 | 0.963 | 1 |
| **Sleep pattern** |  |  |  |  |  |
| Poor | Ref | - | - | - | 32 |
| Intermediate | -0.64 | 0.53 | 0.29, 0.95 | 0.033 | 9 |
| Healthy | -0.90 | 0.41 | 0.22, 0.75 | 0.004 | 0 |
| **Family history of cancer** |  |  |  |  |  |
| No | Ref | - | - | - | 0 |
| Yes | 0.14 | 1.15 | 0.86, 1.54 | 0.355 | 5 |
| **Diabetes** |  |  |  |  |  |
| No | Ref | - | - | - | 0 |
| Yes | 1.13 | 3.10 | 2.05, 4.69 | <0.001 | 40 |
| **High Cholesterol** |  |  |  |  |  |
| No | Ref | - | - | - | 10 |
| Yes | -0.28 | 0.76 | 0.51, 1.13 | 0.170 | 0 |
| **CVD** |  |  |  |  |  |
| No | Ref | - | - | - | 0 |
| Yes | 0.27 | 1.31 | 0.91, 1.87 | 0.145 | 9 |
| **Viral hepatitis** |  |  |  |  |  |
| No | Ref | - | - | - | 0 |
| Yes | 2.82 | 16.83 | 8.92, 31.76 | <0.001 | 100 |
| **Liver disease** |  |  |  |  |  |
| No | Ref | - | - | - | 0 |
| Yes | 2.73 | 15.46 | 8.29, 28.86 | <0.001 | 97 |

Abbreviations: sHR = Subhazard Ratio; CI = Confidence Interval; BMI = Body Mass Index; CVD = Cardiovascular disease

**Supplemental Table 5.** Total score points of the nomogram with corresponding 5-year probability of liver cancer estimated from the multivariable Fine-Gray regression model

| **Total Points** | **5-Year event probability (per 100,000)** |
| --- | --- |
| 0 | 2 |
| 31 | 5 |
| 56 | 10 |
| 80 | 20 |
| 113 | 50 |
| 137 | 100 |
| 162 | 200 |
| 194 | 500 |
| 219 | 1000 |
| 244 | 2000 |
| 277 | 5000 |
| 302 | 10000 |
| 329 | 20000 |
| 369 | 50000 |
